# Gum Acacia–Dexamethasone Combination Attenuates Sepsis-Induced Acute Kidney Injury in Rats via Targeting SIRT1-HMGB1 Signaling Pathway and Preserving Mitochondrial Integrity

**DOI:** 10.3390/ph18081164

**Published:** 2025-08-05

**Authors:** Fawaz N. Alruwaili, Omnia A. Nour, Tarek M. Ibrahim

**Affiliations:** Department of Pharmacology and Toxicology, Faculty of Pharmacy, Mansoura University, Mansoura 35516, Egypt; fawaz2372@hotmail.com (F.N.A.); tarekmoss@yahoo.com (T.M.I.)

**Keywords:** sepsis-associated AKI, LPS, gum acacia, SIRT1, HMGB1, DEX

## Abstract

**Background/Objective:** Sepsis-associated acute kidney injury (SA-AKI) is a substantial contributor to mortality in critically ill patients. This study aimed to investigate the impact of gum acacia (GA) and dexamethasone (DEX) combination on lipopolysaccharide (LPS)-induced SA-AKI in rats. **Methods**: Thirty-six male Sprague Dawley rats were separated into six groups, including the control, GA group, LPS-induced AKI group, DEX + LPS group, GA + LPS group, and GA + DEX + LPS group. AKI was induced in rats using LPS (10 mg/kg, i.p.). GA was administered orally (7.5 g/kg) for 14 days before LPS injection, and DEX was injected (1 mg/kg, i.p.) 2 h after LPS injection. **Results**: LPS injection significantly (*p* < 0.05, vs. control group) impaired renal function, as evidenced through increased levels of kidney function biomarkers, decreased creatinine clearance, and histopathological alterations in the kidneys. LPS also significantly (*p* < 0.05, vs. control group) elevated levels of oxidative stress markers, while it reduced levels of antioxidant enzymes. Furthermore, LPS triggered an inflammatory response, manifested by significant (*p* < 0.05, vs. control group) upregulation of Toll-like receptor 4, myeloid differentiation primary response 88, interleukin-1β, tumor necrosis factor-α, and nuclear factor-κB, along with increased expression of high-mobility group box 1. Administration of GA significantly ameliorated LPS-induced renal impairment by enhancing antioxidant defenses and suppressing inflammatory pathways (*p* < 0.05, vs. LPS group). Furthermore, GA-DEX-treated rats showed improved kidney function, reduced oxidative stress, and attenuated inflammatory markers (*p* < 0.05, vs. LPS group). **Conclusions**: The GA-DEX combination exhibited potent renoprotective effects against LPS-induced SA-AKI, possibly due to their antioxidant and anti-inflammatory properties. These results suggest that the GA-DEX combination could be a promising and effective therapeutic agent for managing SA-AKI.

## 1. Introduction

Sepsis, an inflammatory response, is mediated by the host immune system via interactions between pattern recognition receptors (PRRs) and bacterial lipopolysaccharide (LPS). These interactions result in a streaming of pro-inflammatory mediators, particularly IL-6 and TNF-α [1]. Sepsis-associated acute kidney injury (SA-AKI) is a leading cause of kidney failure [2,3]. LPS-induced AKI is a common model used to investigate SA-AKI [4]. LPS interact with Toll-like receptor 4 (TLR4) which is a PRR expressed in immune and proximal tubule epithelial cells, leading to the activation of inflammatory pathways, T cells, and nuclear factor kappa B (NF-κB), as well as increasing oxidative stress in renal tubule tissues [5].

Evidence indicates that SIRT1, a deacetylase enzyme, plays a crucial role in regulating ROS production in mitochondria in the renal tubule endothelium. Accordingly, it could be a therapeutic target in immune-involved reactions, reducing their negative consequences. SIRT1 deacetylates HMGB1, a damage-associated molecular pattern, leading to attenuation of the inflammatory response and oxidative stress [6]. Accordingly, targeting SIRT1-HMGB1 signaling in the renal cell mitochondria could be of value in controlling LPS-induced sepsis.

Plant-derived agents have attracted considerable attention because they are cost-effective and associated with fewer side effects [7]. Gum acacia (GA) is an exudate with a gummy texture obtained from the umbrella-shaped branches of *Acacia seyal* and *Acacia senegal* [8]. GA has demonstrated significant antioxidant, anti-inflammatory, and antibacterial activity and immune-modulatory effects [9,10,11]. Thus, GA is widely used in medicinal applications. Its antioxidant activity decreases free radicals, which are the main trigger of immune response. Decreasing free radical production will decrease the immune response, attenuating inflammation resulting from such immune response [12]. A number of studies have demonstrated the renoprotective effect of GA against renal diseases and nephrotoxic agents, such as chronic kidney disease [11], gentamicin [13], and cisplatin [14]. Dexamethasone (DEX), a standard anti-inflammatory drug, can attenuate SA-AKI by dysregulating the immune response to PRR-LPS interactions [15,16]. Consequently, this study aimed to elucidate the impact of GA-DEX combination treatment on LPS-induced AKI in rats via enhancing the antioxidant defense system and suppressing inflammation through modulating the SIRT1/HMGB1/NF-κB pathway. To investigate these proposed mechanisms, the effects of the GA and DEX combination on kidney functions, oxidant/antioxidant balance, and pro-inflammatory cytokines were assessed.

## 2. Results

### 2.1. Impact of GA, DEX, and Combination of GA and DEX on LPS-Induced Changes in Body Weight and Kidney Index

There were no significant differences in body weight or kidney index among all groups.

### 2.2. Impact of GA, DEX, and Combination of GA and DEX on LPS-Induced Changes in Kidney Function Biomarkers

**Figure 1:**
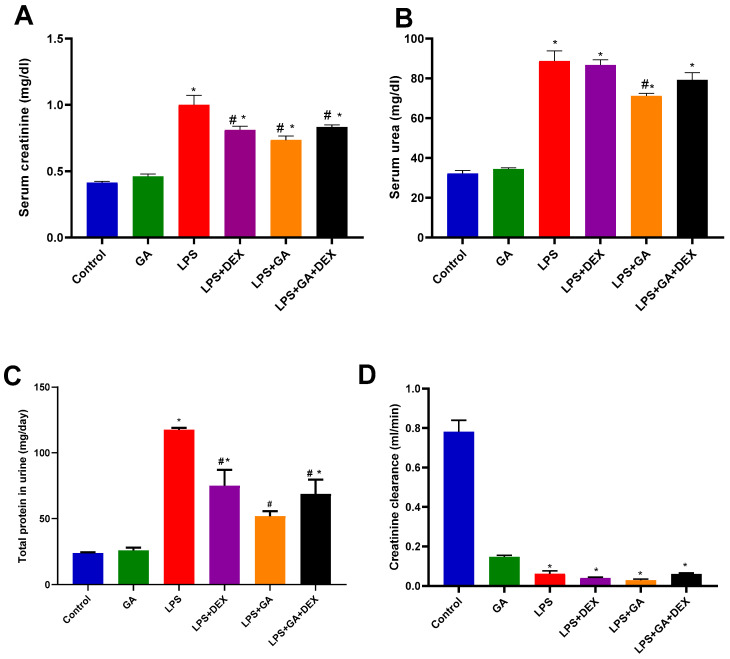
Impact of GA, DEX, and combination of GA and DEX on LPS-induced changes in kidney function biomarkers. (**A**) Serum creatinine, (**B**) serum urea, (**C**) total protein in urine, and (**D**) creatinine clearance. LPS: lipopolysaccharide; DEX: dexamethasone; GA: gum acacia. GA administration started on day 1 and lasted for 14 days. LPS was injected on day 14, and DEX was injected 2 h post LPS injection. Data were expressed as mean ± S.E.M. (n = 6 rats per group). Mean values were compared using one-way ANOVA followed by post hoc Tukey’s multiple comparison test: * *p* < 0.05 (vs. CTR group); ^#^ *p* < 0.05 (vs. LPS group).

LPS injection significantly increased serum creatinine level by 2.4-fold (*p* < 0.05, vs. control group) (Figure 1A). The administration of GA, DEX, and the combination of DEX and GA elicited significant decreases in serum creatinine levels by 26.5%, 18.8%, and 16.7%, respectively (*p* < 0.05, vs. LPS group) (Figure 1A).

Rats injected with LPS demonstrated a significant 2.7-fold increase in serum urea levels (*p* < 0.05, vs. control group) (Figure 1B). Nevertheless, GA administration elicited a significant decrease in serum urea levels of 19.7% (*p* < 0.05, vs. LPS group) (Figure 1B). However, administering DEX and the combination of DEX and GA had no significant effect on serum urea levels (Figure 1B). The decrease in serum urea was more significant in the LPS + GA group than in the LPS + DEX and LPS + GA + DEX groups (*p* < 0.05, Figure 1B).

Furthermore, LPS injection significantly elevated total protein levels in urine by 4.9-fold (*p* < 0.05, vs. control group) (Figure 1C). However, the administration of GA, DEX, and the combination of DEX and GA induced a significant reduction in urine total protein levels of 56%, 36.1%, and 41.5%, respectively (*p* < 0.05, vs. LPS group) (Figure 1C).

LPS injection significantly decreased creatinine clearance levels by 92% (*p* < 0.05, vs. control group) (Figure 1D). However, administration of GA, DEX, and the combination of DEX and GA had no significant impact on creatinine clearance levels (Figure 1D).

### 2.3. Impact of GA, DEX, and Combination of GA and DEX on LPS-Induced Changes in Oxidant/Antioxidant Parameters

**Figure 2:**
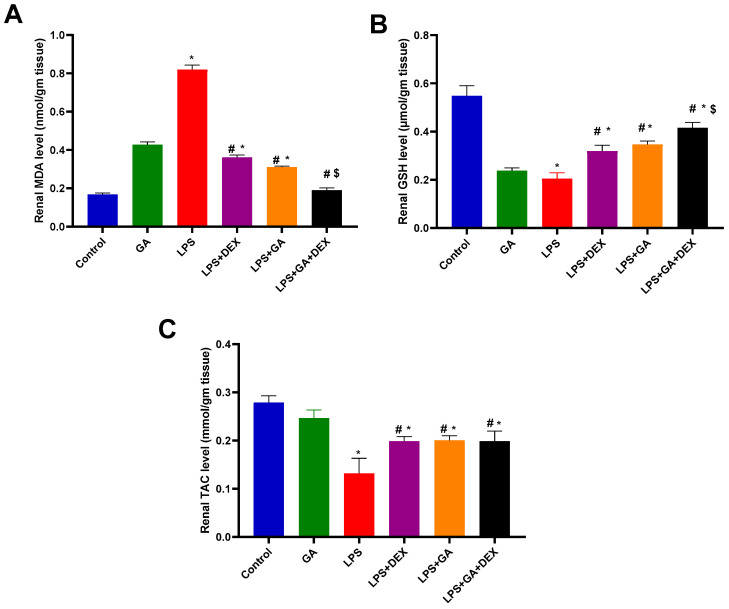
Impact of GA, DEX, and combination of GA and DEX on LPS-induced changes in oxidant/antioxidant parameters. (**A**) Renal MDA content, (**B**) renal GSH content, and (**C**) renal TAC level. LPS: lipopolysaccharide; DEX: dexamethasone; GA: Gum acacia; MDA: malondialdehyde; GSH: glutathione; TAC: total antioxidant capacity. GA administration started on day 1 and lasted for 14 days. LPS was injected on day 14, and DEX was injected 2 h post LPS injection. Data were expressed as means ± S.E.M. (n = 6 rats per group). Mean values were compared using one-way ANOVA followed by post hoc Tukey’s multiple comparison test: * *p* < 0.05, vs. CTR group; ^#^ *p* < 0.05, vs. LPS group; ^$^ *p* < 0.05, vs. LPS + GA and LPS + DEX group.

Injection of LPS elicited a significant 4.9-fold increase in MDA levels in kidney homogenate (*p* < 0.05, vs. control group) (Figure 2A). Nonetheless, the administration of GA, DEX, and the combination of DEX and GA induced a significant decline in MDA levels in kidney homogenate of 62%, 56%, and 77%, respectively (*p* < 0.05, vs. LPS group) (Figure 2A). The mediated effect of the combination of DEX and GA on MDA content was more significant than that of LPS+ GA and LPS + DEX (*p* < 0.05, Figure 2A).

Furthermore, rats injected with LPS demonstrated a significant decrease in GSH content in kidney homogenate of 62.6% (*p* < 0.05, vs. control group) (Figure 2B). Nevertheless, the administration of GA, DEX, and the combination of DEX and GA induced significant 1.7-fold, 1.5-fold, and 2-fold increases in GSH content, respectively (*p* < 0.05, vs. LPS group) (Figure 2B). The mediated effect of the combination of DEX and GA on GSH content was more significant than that of LPS + GA and LPS + DEX (*p* < 0.05, Figure 2B).

In comparison to the control group, LPS injection caused a significant decline in TAC levels in kidney homogenate 52.6% (*p* < 0.05, vs. control group) (Figure 2C). Nevertheless, the administration of GA, DEX, and the combination of DEX and GA induced significant 1.52-fold, 1.5-fold, and 1.5-fold increases in TAC levels in kidney homogenate, respectively (*p* < 0.05, vs. LPS group) (Figure 2C). 

### 2.4. Impact of GA, DEX, and Combination of GA and DEX on LPS-Induced Changes in Inflammatory Markers

**Figure 3:**
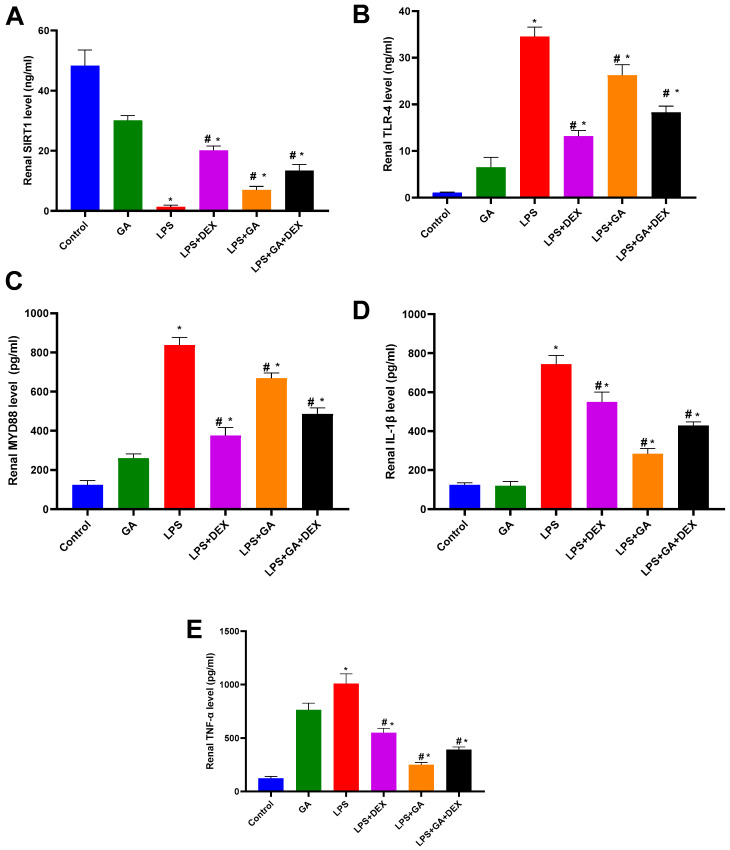
Impact of GA, DEX, and combination of GA and DEX on LPS-induced changes in inflammatory markers. (**A**) Renal SIRT1 level, (**B**) renal TLR4 level, (**C**) renal MYD88 level, (**D**) renal IL-1β level, and (**E**) renal TNF-α level. LPS: lipopolysaccharide; DEX: dexamethasone; GA: Gum acacia; SIRT1: Silent information regulator 2-related enzyme 1; TLR4: Toll-like receptor 4; TNF-α: tumor necrosis factor α; IL-1β: interleukin -1β; MYD88: myeloid differentiation primary response 88. GA administration started on day 1 and lasted for 14 days. LPS was injected on day 14, and DEX was injected 2 h post LPS injection. Data were expressed as means ± S.E.M. (n = 6 rats per group). Mean values were compared using one-way ANOVA followed by post hoc Tukey’s multiple comparison test: * *p* < 0.05, vs. CTR group; ^#^ *p* < 0.05, vs. LPS group.

Rats injected with LPS displayed a significant decrease in SIRT1 levels in kidney homogenate by 97 % (*p* < 0.05, vs. control group) (Figure 3A). Nevertheless, the administration of GA, DEX, and the combination of DEX and GA elicited significant 5.2-fold, 15-fold, and 10-fold increases in SIRT1 levels, respectively (*p* < 0.05, vs. LPS group) (Figure 3A). The SIRT1 level elevation was more significant in the LPS+ DEX group than in the LPS + GA and LPS + GA + DEX groups (*p* < 0.05, Figure 3A).

Moreover, LPS injection significantly increased TLR4 levels in kidney homogenate by 33-fold (*p* < 0.05, vs. control group) (Figure 3B). Nonetheless, the administration of GA, DEX, and the combination of DEX and GA induced significant decreases in kidney tissue TLR4 levels of 24%, 62%, and 47%, respectively (*p* < 0.05, vs. LPS group) (Figure 3B). The reduction in TLR4 levels was greater in the LPS + DEX group than in the LPS + GA and LPS + GA + DEX groups (*p* < 0.05, Figure 3B).

LPS injection significantly increased MYD88 levels in kidney homogenate, raising them 6.8-fold (*p* < 0.05, vs. control group) (Figure 3C). However, the administration of GA, DEX, and the combination of DEX and GA resulted in a significant decline in MYD88 levels of 20%, 55%, and 42%, respectively (*p* < 0.05, vs. LPS group) (Figure 3C). The reduction in MYD88 levels was greater in the LPS + DEX group than in the LPS + GA and LPS + GA + DEX groups (*p* < 0.05, Figure 3C). Additionally, the rats injected with LPS revealed a significant elevation in IL-1β levels of 29% (*p* < 0.05, vs. control group) (Figure 3D). The administration of GA, DEX, and the combination of DEX and GA elicited a significant decrease in IL-1β levels of 62%, 26%, and 42%, respectively (*p* < 0.05, vs. LPS group) (Figure 3D). The reduction in IL-1β levels was more pronounced in the LPS+ GA group than in the LPS + DEX and LPS + GA + DEX groups (*p* < 0.05, Figure 3D).

The rats injected with LPS exhibited a significant 8.1-fold elevation in TNF-α level (*p* < 0.05, vs. control group) (Figure 3E). Yet, the administration of GA, DEX, and the combination of DEX and GA induced significant decreases in TNF-α levels of 75.1%, 45.4%, and 61.26%, respectively (*p* < 0.05, vs. LPS group) (Figure 3E). The decrease in TNF-α level was greater in the LPS + GA group than in the LPS + DEX and LPS + GA + DEX groups (*p* < 0.05, Figure 3E).

### 2.5. Impact of GA, DEX, and Combination of GA and DEX on LPS-Induced Histopathological Irregularities in Kidney Tissues

**Figure 4:**
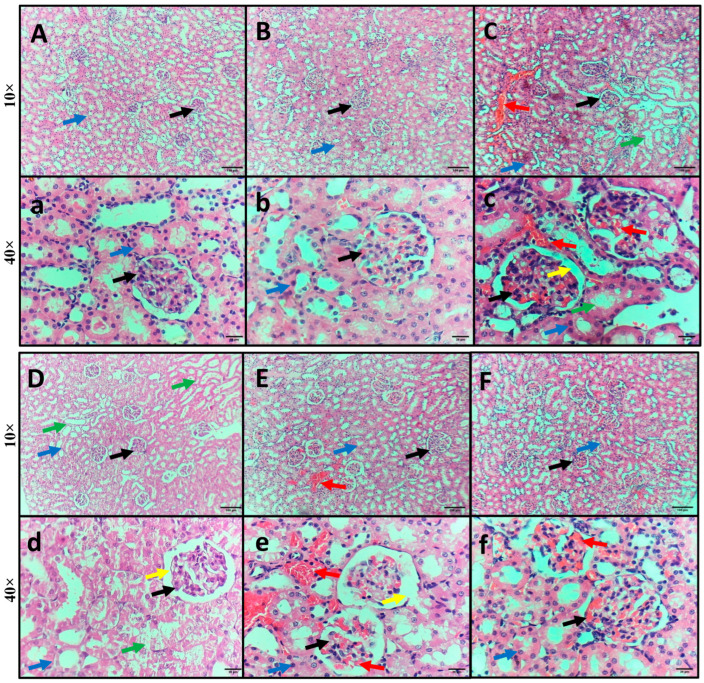
Impact of GA, DEX, and combination of GA and DEX on LPS-induced histopathological irregularities in kidney tissues. (**A**–**F**) Representative microscopic pictures of H&E-stained kidney specimens of (**A**,**a**) control, (**B**,**b**) GA, (**C**,**c**) LPS, (**D**,**d**) LPS + DEX, (**E**,**e**) LPS + GA, and (**F**,**f**) LPS + DEX + GA group. Arrow indications: black arrow: glomeruli; blue arrow: renal tubules; yellow arrow: widened Bowman’s space; green arrow: swelling or degeneration of tubular epithelial cells; red arrow: glomerular or intratubular hemorrhage.

Kidney specimens from the control and GA groups showed normal kidney structure, with normal-sized glomeruli surrounded by Bowman’s space and normal renal (proximal and distal) tubules (Figure 4A,B). However, kidney specimens from the rats injected with LPS exhibited intratubular and glomerular hemorrhage, swelling and degeneration of tubular epithelial cells, glomerular shrinkage, and widened Bowman’s space (Figure 4C). Kidney specimens isolated from the rats injected with LPS and DEX exhibited mild swelling and degeneration of tubular epithelial cells and minimal glomerular shrinkage with widened Bowman’s space (Figure 4D). Kidney specimens isolated from the rats injected with LPS and administered GA showed mild intratubular and glomerular hemorrhage and minimal glomerular shrinkage with widened Bowman’s space (Figure 4E). Kidney specimens isolated from the rats injected with LPS and DEX and administered GA revealed restoration of normal kidney structure, with minimal hemorrhage (Figure 4F).

### 2.6. Impact of GA, DEX, and Combination of GA and DEX on LPS-Induced Changes in NF-κB and HMGB1 Expression

**Figure 5:**
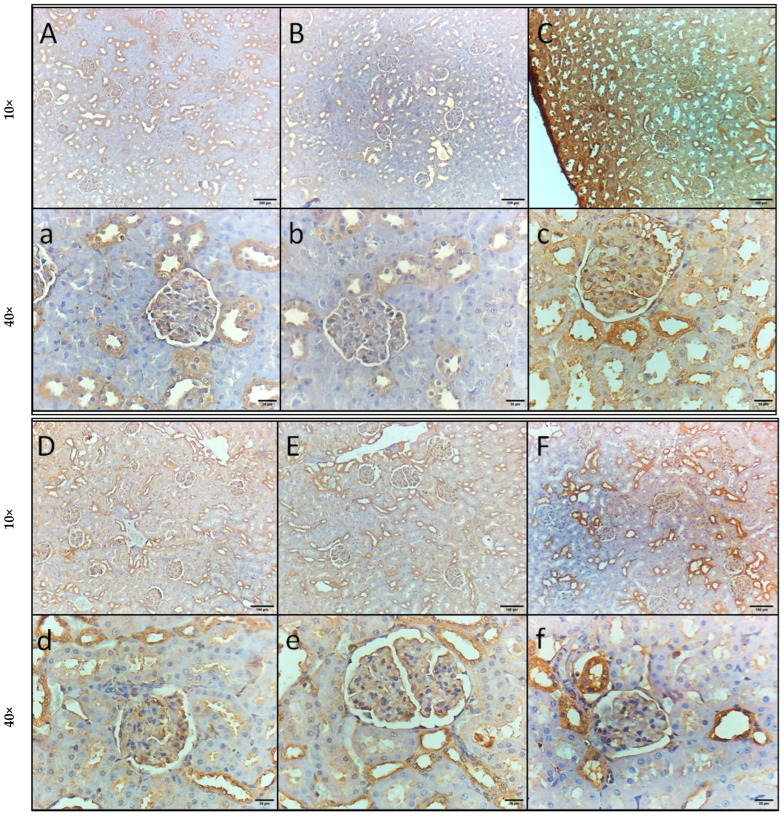

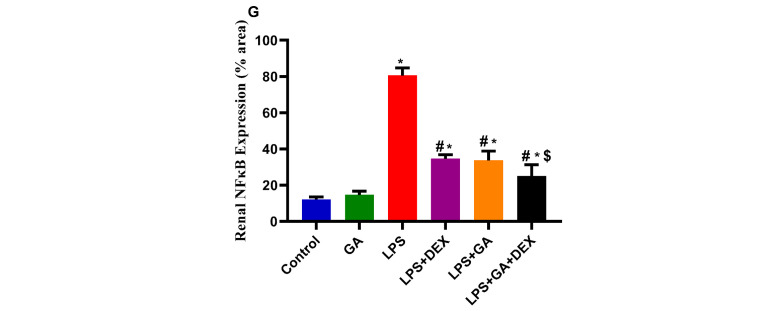
Impact of GA, DEX, and combination of GA and DEX on LPS-induced changes in nuclear factor-κB (NF-κB) expression. (**A**,**a**) Control group, (**B**,**b**) GA group, (**C**,**c**) LPS group, (**D**,**d**) LPS + DEX group, (**E**,**e**) LPS+ GA group, (**F**,**f**) LPS+ DEX+ GA group, and (**G**) renal NF-κB expression. LPS: lipopolysaccharide; DEX: dexamethasone; GA: gum acacia; NF-κB: nuclear factor-κB. GA administration started on day 1 and lasted for 14 days. LPS was injected on day 14, and DEX was injected 2 h post LPS injection. Data were expressed as means ± S.E.M. (n = 6 rats per group). Mean values were compared using one-way ANOVA followed by post hoc Tukey’s multiple comparison test: * *p* < 0.05, vs. CTR group; ^#^ *p* < 0.05, vs. LPS group; ^$^ *p* < 0.05, vs. LPS + GA and LPS + DEX group.

NF-κB immunostaining of kidney sections revealed normal kidney structure with mild immunoreactivity in podocytes and some renal tubular cells in the control and GA groups (Figure 5A,B). The LPS-treated group showed high immunoreactivity in all podocytes and renal tubular cells (Figure 5C). However, kidney specimens isolated from the rats injected with LPS and administered GA, as well as the rats injected with LPS and DEX, showed moderate immunoreactivity in all podocytes and some renal tubular cells (Figure 5D,E). Furthermore, kidney specimens isolated from the rats injected with LPS and DEX and administered GA showed mild immunoreactivity in all podocytes and some tubular cells (Figure 5F).

NF-κB expression was significantly elevated in the rats injected with LPS by 6.6-fold (*p* < 0.05, vs. control group) (Figure 5G). However, the administration of GA, DEX, and the combination of DEX and GA elicited a significant decline in NF-κB expression of 57%, 58%, and 69%, respectively (*p* < 0.05, vs. LPS group) (Figure 5G). The reduction in NF-κB expression was greater in the LPS + GA + DEX group than in the LPS + DEX and LPS + GA groups (*p* < 0.05, Figure 5G).

**Figure 6:**
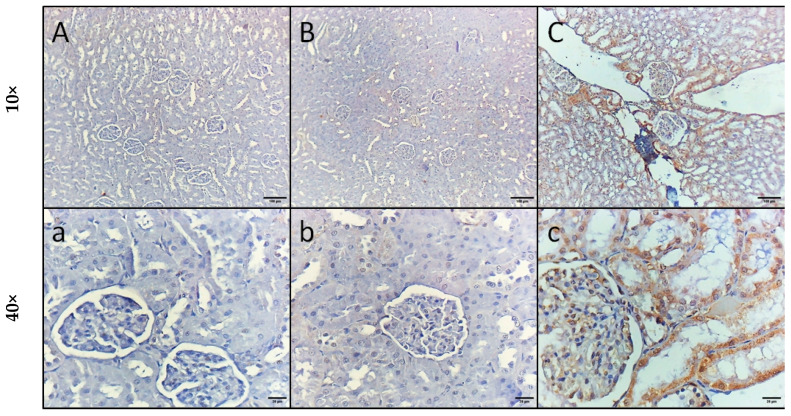

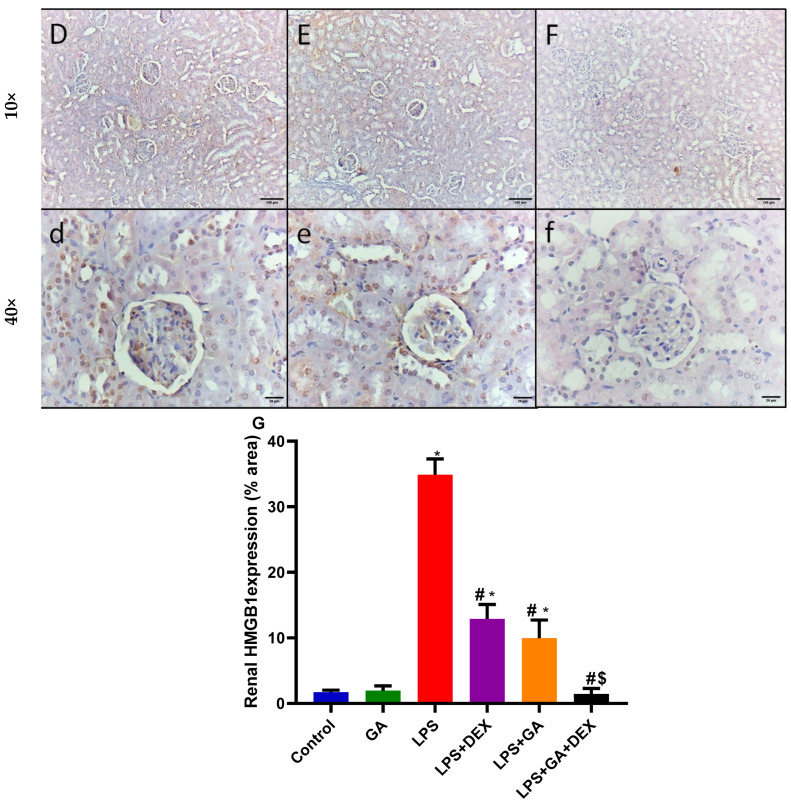
Impact of GA, DEX, and combination of GA and DEX on LPS-induced changes in HMGB1 expression. (**A**,**a**) Control group, (**B**,**b**) GA group, (**C**,**c**) LPS group, (**D**,**d**) LPS + DEX group, (**E**,**e**) LPS + GA group, (**F**,**f**) LPS + DEX + GA group, and (**G**) renal HMGB1 expression. LPS: lipopolysaccharide; DEX: dexamethasone; GA: gum acacia; HMGB1: high-mobility group box1. GA administration started on day 1 and lasted for 14 days. LPS was injected on day 14, and DEX was injected 2 h post LPS injection. Data were expressed as means ± S.E.M. (n = 6 rats per group). Mean values were compared using one-way ANOVA followed by post hoc Tukey’s multiple comparison test: * *p* < 0.05, vs. CTR group; ^#^ *p* < 0.05, vs. LPS group; ^$^ *p* < 0.05, vs. LPS + GA and LPS + DEX group.

Kidney sections from the rats injected with LPS exhibited a significant 6.6-fold increase in HMGB1 expression in contrast to the control group (*p* < 0.05, vs. control group) (Figure 6G). The administration of GA, DEX, and the combination of DEX and GA elicited a significant decrease in HMGB1 expression of 63%, 71.1%, and 96%, respectively (*p* < 0.05, vs. LPS group) (Figure 6G). The decrease in HMGB1 expression was greater in the LPS + GA + DEX group than in the LPS + DEX and LPS + GA groups (*p* < 0.05, Figure 6G).

## 3. Discussion

Sepsis is one of the most common causes of death among hospitalized patients in critical care units. It induces multi-organ failure, resulting in high mortality [16,17,18]. Sepsis-associated acute kidney injury (SA-AKI) is the leading form of acute kidney failure and a frequent complication in hospitalized and critically ill patients [19,20]. Lipopolysaccharide (LPS) is a component of the outer membrane of Gram-negative bacteria [4]. LPS interacts with Toll-like receptor 4 (TLR4), which is expressed in the immune and proximal tubule epithelial cells, activating inflammatory pathways and resulting in nuclear factor kappa B (NF-κB) activation, the production of pro-inflammatory cytokines, including tumor necrosis factor (TNF)-α, and increased production of reactive oxygen species (ROS) and recruitment of neutrophils, macrophages, and endothelial cells. These outcomes result in renal hypoperfusion, low blood pressure, and a gradual decrease in kidney function [21,22]. ROS augment apoptotic cell death, cellular detachment, and dysfunction in proximal tubules [23]. Additionally, TLR4 augments MyD88 expression, activating the NF-κB signaling pathway and inducing inflammatory factor release [24]. Ultimately, these alterations result in structural and functional renal injury.

Our results indicate that LPS administration resulted in impairment of renal function, indicated by increases in serum urea and creatinine levels and total protein levels in urine. Conversely, LPS administration resulted in a significant decrease in creatinine clearance, which reflects the rate of glomerular filtration, indicating renal dysfunction and kidney injury. Histopathologically, the LPS-treated group showed swelling and degeneration of tubular epithelial cells and glomerular shrinkage with widened Bowman’s space. However, GA administration for 14 days resulted in attenuation of SA-AKI, as indicated by improvements in renal function. Moreover, kidney sections from the rats treated with GA showed mild histopathological changes. These findings highlight the potential renoprotective effect of GA treatment in SA-AKI. These findings align with those of previous studies [14,25].

Oxidative stress is strongly linked with the development and progression of SA-AKI [26]. Former studies demonstrated that LPS administration results in mitochondrial dysfunction, leading to an imbalance between ROS and the antioxidant system, resulting in oxidative stress [2,27]. In the kidney, LPS administration induces excess production of ROS that destroy the membrane proteins, lipids, and DNA in kidney parenchyma, directly damaging the cells. Additionally, the presence of excessive ROS during kidney injury depletes antioxidant enzymes, including superoxide dismutase (SOD) and glutathione (GSH) [28]. LPS administration significantly decreased TAC and GSH levels compared to the control group, indicating a compromised antioxidant defense system in LPS-induced AKI. Conversely, LPS significantly increased the MDA content, enhancing lipid peroxidation and consequently oxidative stress. GA administration for 14 days elicited an antioxidant effect, manifested by significant increases in GSH and TAC levels and a marked reduction in MDA levels compared to the LPS group. These findings are in accordance with those of a former study which showed that GA decreases oxidative stress in diabetic rats with adenine-induced chronic kidney disease [29]. Moreover, in humans suffering from oxidative stress due to chronic inflammation, GA is able to increase TAC levels and decrease MDA levels [30].

High-mobility group box1 (HMGB1) and Sirtuin 1 (SIRT1) signaling pathways are evolutionarily conserved, promoting the maintenance of homeostasis, and their interaction directly regulates inflammatory responses [6]. HMGB1, a widely available protein, is a potential inflammatory cytokine associated with many kidney diseases. HMGB1 binds to TLR4, activating pro-inflammatory signaling pathways [31,32]. It is considered a mediator of early inflammation and a late mediator of lethal sepsis. Moreover, HMGB1 is regarded as a crucial predictor of organ dysfunction and outcome in patients with severe sepsis [33]. Silent information regulator 2-related enzyme 1 (sirtuin 1, SIRT1) is a nicotinamide adenine dinucleotide (NAD)-dependent class III histone deacetylase. It has an important role in controlling cellular oxidative stress response and inflammatory disorders, including kidney disease, by regulating the production of pro-inflammatory cytokines through NF-κB acetylation [34,35]. SIRT1 activation results in attenuation of mitochondrial dysfunction and oxidative stress [36]. It has a nephroprotective impact in a variety of renal diseases, including AKI. Its effect can be mediated by reducing oxygen free radicals, attenuating apoptosis, and stabilizing mitochondrial function [37]. SIRT1-mediated deacetylation profoundly influences several biological processes, including oxidative stress and inflammation. It exerts antioxidant and anti-inflammatory properties [38].

LPS-TLR4 interaction results in the translocation of HMGB1 from the cell nucleus, where it acts as a DNA chaperone protein, to the cytoplasm to enhance cell autophagy and eliminate injured cells. After its active secretion or passive release to the outside of the cell, it interacts as a damage-associated molecular pattern (DAMP) that stimulates immune cells, leading to pro-inflammatory cytokine release and a dysregulated inflammatory response [39]. HMGB1 activity is controlled by a balanced acetylation–deacetylation and methylation process. Hyperacetylation of HMGB-1 increases the inflammatory response. The process of HMGBI acetylation requires the oxidation of three cysteine residues located in the DNA-binding domains [40]. On the contrary, HMGB-1 deacetylation suppresses its pro-inflammatory activity. This deacetylation process depends on the activity of SIRT1, which is an NAD +-dependent histone deacetylase [41]. LPS promote the hyperacetylation of HMGB1, resulting in its secretion from the nucleus and enabling its release from the cell. However, SIRT1 deacetylates HMGB1, causing HMGB1 to remain in the nucleus and reducing its cytoplasmic translocation. It has been revealed that SIRT1-mediated HMGB1 deacetylation suppresses SA-AKI [42].

The TLR4-NF-κB pathway plays a crucial role in modulating mitochondria-related oxidative damage [43]. LPS activates the TLR4-MyD88-dependent signaling pathway. MyD88 recruits to TLR4, mediating the phosphorylation of the IKK complex and resulting in nuclear NF-κB translocation and subsequent expression of pro-inflammatory genes, including TNF-α [44]. NF-κB, a nuclear transcription factor, is involved in inflammation and oxidative stress [45]. NF-κB translocates to the nucleus, enhancing aggravation of the inflammatory response and oxidative stress damage and subsequently causing kidney damage [46]. Previous studies have demonstrated that NF-κB expression is increased in the kidney tissue of septic AKI rats [46].

Interestingly, our study demonstrated an increase in the protein expression of IL-1β, MYD88, TLR4, and TNF-α and a significant decrease in SIRT1 expression in the kidney homogenate of rats injected with LPS. Additionally, NF-κB and HMGB1 immunostaining of renal tissues indicated high immunoreactivity in rats with LPS-induced AKI. These results are in line with those of previous studies [47,48,49].

Nevertheless, GA administration for 14 consecutive days prior to LPS administration resulted in SA-AKI attenuation, as indicated by marked decrease in IL-1β, MYD88, TLR4, and TNF-α protein expressions. Additionally, NF-κB and HMGB1 immunostaining of renal tissues showed low immunoreactivity in the rats administered GA, indicating the anti-inflammatory effect of GA. Moreover, the administration of GA resulted in a remarkable increase in SIRT1 expression compared to the LPS group. This significant increase indicates the potential of GA to exert protective effects against renal injury. GA’s antioxidant effect may be mediated by SIRT1 activation. The anti-inflammatory effect might be elucidated by GA’s antioxidant effect and potent ability to decrease ROS, which are the main inducers of inflammation. In addition, GA has a potent suppressive effect on NF-κB expression, which is the main transcription factor for TNF-α.

In our study, dexamethasone (DEX) was used as standard drug for the treatment of sepsis. DEX, a long-acting glucocorticoid, has anti-inflammatory and immunosuppressive effects [50]. It inhibits the process of chemotaxis by inhibiting the cytoplasmic protein immunophilin in macrophages and neutrophils [51]. Clinically, a low “physiologic dose” of glucocorticoid has been recommended in the treatment of septic shock, improving survival [52]. It has been shown that early DEX use alleviated sepsis-induced AKI, indicated by decreases in serum creatinine and BUN [53]. Moreover, a low dose of DEX could increase glucocorticoid receptor-α levels and decrease plasma levels of TNF-α and IL-1β in the kidney, alleviating sepsis-induced kidney injury [54]. A study by Tsao et al. indicated that a low dose of DEX mitigated renal dysfunction in conscious rats when administered concurrently with LPS [55]. DEX use resulted in a marked improvement in the markers that indicate SA-AKI.

The combined impact of GA and DEX also resulted in a significant improvement in the inflammatory status of the kidney and kidney function. The effect of the combination on oxidative stress markers was superior to both GA alone and DEX alone. Similar results indicating the superiority of the combined effect were obtained when comparing the levels of HMGB1 and NF-κB following the administration of either GA alone or DEX alone.

Concerning SIRT1, TLR-4, MYD88, and TNF-α, the effect of DEX was superior to the effect of GA alone or the combination of GA and DEX

Concerning improvement in the level of the inflammatory markers IL-1β and TNF-α, the effect of GA was superior to the effect of DEX alone or the combination of GA and DEX. Immunohistopathological examination revealed that LPS administration caused a significant increase in NF-κB expression, while GA administration, DEX administration, and their combined effect caused significant improvements compared to the effect of LPS. GA administration alone before LPS administration significantly decreased the LPS-induced increase in renal HMGB1, and a significant decrease in HMGB1 expression also resulted from DEX administration. The combined effect of GA and DEX nearly normalized the increased expression of HMGB1 induced by LPS.

Dutta, M. and Paul, G. (2021) stated that GA protects rat liver mitochondria against damage induced by bisphenol via an antioxidant effect [56]. SIRT1 activity is dependent on cellular NAD+; thus, abundant cellular NAD+ directly enhances SIRT1 activity [57,58]. Mitochondria are the site of NAD+ synthesis during NADH oxidation via the activity of the electron transport chain (ETC), which is the major contributor to the oxidation of NADH into NAD^+^ [59]. Accordingly, the preservation of mitochondrial integrity by GA through its antioxidant effect may demonstrate the GA-induced renoprotective effect against sepsis-induced AKI. Interestingly, Han et al. (2003) stated that polysaccharides are possible TLR4 ligands [60]. LPS are ligands of TLR4, as mentioned in a previous study [61]. The lipid moiety of LPS is essential for the pathological consequences of the TLR4/LPS interaction. Accordingly, there may be a type of competitive interaction between the GA polysaccharides and LPS for TLR4 binding sites, inhibiting innate immunity and stimulating the effect of the TLR4/LPS complex. This may offer an additional explanation for the mitochondrial integrity preservation induced by GA.

It was reported that DEX administration attenuated SA-AKI by enhancing mitochondrial function. The ROS-decreasing activity and its inhibitory effect on NADPH oxidase (NOX)-2-dependent ROS generation could explain the beneficial effect of DEX in preserving good mitochondrial function in LPS-induced sepsis [62]. T.J.J. Schirris et al. (2017) also examined DEX’s effect on mitochondrial function, stating that its slight intracellular acidifying effect, which reverses LPS-induced alkalinity, could result in the upregulation of mitochondrial function [63]. Accordingly, DEX also could enhance the expression of SIRT1, leading to increased deacetylation of HMGB1. These consequences may illustrate the superiority of the GA-DEX combination effect in suppressing sepsis-induced AKI.

## 4. Material and Methods

### 4.1. Drugs and Chemicals

Lipopolysaccharides (LPS) (Escherichia coli, serotype O111: B4, and phenol-purified) were obtained from Sigma Aldrich Chemical Co., St. Louis, MO, USA. GA acacia (GA) was obtained in the form of 100% pure HASHAB-grade Acacia Senegal E−414 powder from the UAE. Dexamethasone (DEX) was obtained in ampoules from Amriya Company, Alexandria, Egypt.

### 4.2. Animals

Thirty-six male Sprague Dawley rats, weighing 180 ± 20 g, were obtained from the Medical Experimental Research Center at the Faculty of Medicine, Mansoura University, Egypt. The rats were allowed a one-week acclimatization period prior to the experiment.

Throughout the experimental period, the animals were maintained under constant nutritional and environmental conditions at room temperature with a 12 h on/off light schedule. Animals were handled according to the rules of the Committee of Ethics of Scientific Research, Faculty of Pharmacy, Mansoura University, Egypt. These practices are in agreement with the principles of Laboratory Animal Care (NIH publication no. 85-23, revised 1985). The experimental protocol was approved by the Animal Care and Use Committee of Mansoura University (MU-ACUC), Egypt (MU-ACUC PHARM.R.MS. 23.06.16).

### 4.3. Induction of AKI

AKI was induced in rats via intraperitoneal injection of 10 mg/kg lipopolysaccharides (LPS) dissolved in 0.9% saline [64]. Gum acacia (GA) (7.5 g/kg) was dissolved in distilled water and administered orally for 14 days before LPS injection, and the animals were sacrificed 24 h after LPS injection [65]. Dexamethasone (DEX) (1 mg/kg, i.p.) was injected 2 h after LPS injection [66].

### 4.4. Experimental Protocol and Sample Collection

The animals were allocated into six groups, as described below:

Control group (n = 6 rats): The rats received distilled water for 14 days and were i.p. administered 0.9% saline on day 14. They received no LPS and no drugs. 

GA group (n = 6 rats): The rats received GA (7.5 g/kg) orally for 14 days. 

LPS group (n = 6 rats): The rats received LPS injection (10 mg/kg, i.p.) on day 14 and received no drugs. 

GA + LPS group (n = 6 rats): The rats received GA (7.5 g/kg) orally for 14 days, followed by LPS injection (10 mg/kg) on day 14.

DEX + LPS group (n = 6 rats): The rats were injected with 10 mg/kg LPS on day 14, followed by DEX (1 mg/kg, i.p.) 2 h after LPS injection.

GA + DEX +LPS group (n = 6 rats): The rats received GA (7.5 g/kg) orally for 14 days, followed by LPS injection (10 mg/kg, i.p.) on day 14. They then received DEX (1 mg/kg, i.p.) 2 h after LPS injection. 

GA was administered from day 1 to day 14. LPS was injected on day 14, and DEX was injected 2 h post LPS injection on day 14.

On day 14, all rats were retained in metabolic cages individually so we could collect their urine for 24 h. The urine samples were centrifuged at 3000 rpm for 15 min and stored at −80 °C. Urine samples were used for assessing total protein and creatinine. 

### 4.5. Sample Preparation

At the end of the experimental period, the animals were anesthetized with thiopental sodium (40 mg/kg, i.p.). Blood samples were collected from the retro-orbital sinus and then centrifuged at 3000× *g* to separate the serum, which was stored at −80 °C for the analysis of blood urea nitrogen and creatinine levels. The kidneys were harvested and weighed to calculate the kidney index.

**Figure 7:**
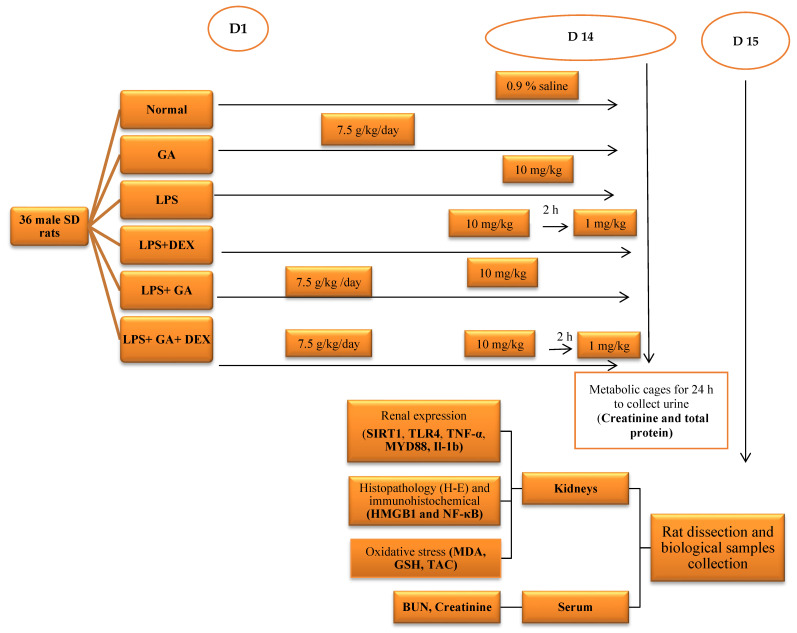
Schematic diagram for the experimental protocol. Abbreviations: Il-1b: interleukin-1b; TNF-α: tumor necrosis factor α; MYD88: myeloid differentiation primary response 88; NF-κB: nuclear factor-κB; MDA: malondialdehyde; GSH: reduced glutathione; BUN: blood urea creatinine; HMGB1: high-mobility group box 1; SIRT1: sirtuin 1; TNF-α: tumor necrosis factor-α; TAC: total antioxidant; DEX: dexamethasone; GA: Gum acacia.

The left kidney was fixed in 10% buffered formalin for histopathological and immunohistochemical examination. The right kidney was isolated and homogenized with a mini handheld homogenizer (Omni International, Kennesaw, GA, USA) to prepare a 10% (*w*/*v*) solution in PBS (pH 7.5), followed by centrifugation at 5000 rpm and 4 °C for 15 min. The supernatants were collected for the assessment of oxidative/antioxidant parameters and levels of sirtuin-1 (SIRT1), Toll-like receptor 4 (TLR4), myeloid differentiation primary response 88 (MYD88), interleukin-1β (IL-1β), and tumor necrosis factor α (TNF-α), using an enzyme-linked immunosorbent assay (ELISA). The experimental design is illustrated in Figure 7.

### 4.6. Assessment of Biological Marker Levels in Serum and Tissue Samples

Kidney Function Biomarkers: Commercial assay kits obtained from Spinreact, Girona, Spain, were used to assess serum creatinine (Cr), serum urea levels, and urinary Cr and total protein, following the manufacturer’s instructions. Serum and urine Cr concentrations were used to calculate creatinine clearance (Ccr), which reflects glomerular filtration. Ccr was determined using the formula Ccr (mL/min) = [urinary Cr (mg/dL) × urine volume (mL) × 1440/serum Cr (mg/dL)], where 1440 is the number of minutes per day.

Oxidative/Antioxidant Parameters: The levels of malondialdehyde (MDA), reduced glutathione (GSH), and total antioxidant capacity (TAC) were measured in kidney homogenate using commercial assay kits from Biodiagnostic Co., Giza, Egypt, in accordance with the manufacturer’s guidelines.

Inflammation Biomarkers: Sirtuin-1 (SIRT1) (BT LABORATORY, Beijing, China, E1145Ra), tumor necrosis factor α (TNF-α) (CUSABIO, Houston, TA, USA, CSB-E11987r), Toll-like receptor 4 (TLR4) (CUSABIO, Houston, TA, USA, CSB-E15822r), myeloid differentiation primary response 88 (MYD88) (Assay Genie, Dublin, Ireland, RTFI01303), and interleukin-1β (IL-1β) (CUSABIO, Houston, TA, USA, CSB-E08055r) were quantified in kidney homogenate using ELISA kits according to the manufacturer’s instructions. The assay utilized a double-antibody sandwich ELISA method, and the optical density (O.D.) of the final product was measured with a spectrophotometer at 450 nm using an ELISA microplate reader (BioTek Instruments ELx800, Winooski, VT, USA).

### 4.7. Histopathological and Immunohistochemical Examination

Isolated left kidney tissues were collected, fixed in 10% formalin, embedded in paraffin wax, sectioned, and stained with hematoxylin–eosin (H&E) for histopathological analysis. The examinations were conducted using light microscopy (Leica Imaging Systems, Cambridge, UK). The pathologist performing the histopathological examination was blinded to the treatment groups.

The renal content of high-mobility group box 1 (HMGB1) and nuclear factor-κB (NF-κB) was evaluated using immunohistochemistry techniques. Briefly, antigen retrieval was carried out after deparaffinization and rehydration. The sections were incubated overnight at 4 °C with a rat polyclonal antibody (Thermo Scientific, Rockford, IL, USA). After washing, the samples were treated with a goat anti-rat secondary antibody (Genemed Biotechnologies, Torrance, CA, USA)for 2 h at room temperature and visualized using diaminobenzidine. The sections were subsequently analyzed under a light microscope (Leica, Cambridge, UK). Appropriate controls were performed to rule out background or non-specific binding [67]. Using ImageJ software version 1.51r (NIH, Bethesda, MD, USA), the positively stained areas were identified blindly, and the mean of six readings from the left renal sections was calculated for each rat.

### 4.8. Statistical Analysis

Data are presented as the means ± standard error of the mean (S.E.M). Statistical analysis and graphing were conducted using Graph Pad Prism software (Graph Pad Software Inc. V 8.4.2, San Diego, CA, USA). Differences were considered significant at *p* < 0.05. Comparisons between groups were evaluated using one-way analysis of variance (ANOVA), followed by Tukey’s multiple comparison test as a post hoc analysis.

## 5. Conclusions

Collectively, our findings indicate that the gum acacia and DEX combination could ameliorate LPS-induced AKI possibly by enhancing the antioxidant defense system and suppressing inflammation. The combination of GA and DEX could restore the imbalance between oxidant/antioxidant parameters and induce a significant increase in GSH and a significant decrease in MDA, NF-κB, and HMGB1 expression, indicating that GA-DEX combination therapy could be a promising strategy for managing LPS-induced AKI.

### The Limitations of This Study

The current study aimed to elucidate the renoprotective impact of gum acacia on LPS-induced AKI, according to the good response to DEX, through comparison with a group that did not receive prior gum acacia as a protective remedy. Further research on molecular mechanisms using transgenic knockout rats lacking SIRT1 is required and will be undertaken as soon as the research group can afford such experimental animals.

## Data Availability

Data presented in this study is contained within the article. Further inquiries can be directed to the corresponding author.
